# Influence of food preparation behaviors on 5-year weight change and obesity risk in a French prospective cohort

**DOI:** 10.1186/s12966-018-0747-4

**Published:** 2018-11-26

**Authors:** Caroline Méjean, Aurélie Lampuré, Wendy Si Hassen, Séverine Gojard, Sandrine Péneau, Serge Hercberg, Katia Castetbon

**Affiliations:** 1 MOISA, Univ Montpellier, INRA, CIRAD, CIHEAM-IAMM, Montpellier SupAgro, Montpellier, France; 2 Université Paris 13, Sorbonne Paris Cité, Equipe de Recherche en Epidémiologie Nutritionnelle, Centre de Recherche en Epidémiologies et Biostatistiques, Inserm (U1153), Inra (U1125), Cnam, F-93017 Bobigny, France; 3 INRA (USC 1429), Centre Maurice Halbwachs, CNRS, EHESS, ENS, PSL Research University (UMR 8097), F75014 Paris, France; 4 Université Libre de Bruxelles (ULB), Ecole de Santé Publique, Route de Lennik 808, CP 598, B-1070 Bruxelles, Belgium

**Keywords:** Food preparation, Cooking practices, Weight change, Cooking skills, Diet

## Abstract

**Background:**

Food preparation behaviors may markedly determine dietary intake and consequently influence weight status. However, the few available studies have found equivocal results. No study has prospectively investigated the association between food preparation behaviors and weight change over time. We estimated the associations of food preparation behaviors with the 5-year relative weight change and the risk of developing obesity in 12,851 French adults participating in the NutriNet-Santé cohort study. The mediating effect of dietary intake was also addressed.

**Methods:**

Frequency and time for meal preparation, cooking skills, preparation from scratch, kitchen equipment, cooking enjoyment, willingness to cook better/more frequently and dietary intake were assessed at baseline using web-based questionnaire and 24 h records, respectively. Self-reported anthropometric data were collected using questionnaire, at baseline and after 5 years of follow-up. Associations of such behaviors with 5-year relative weight change and the mediation analyses were assessed through multivariate linear regression models, and obesity risk was analyzed with logistic regression, stratified by sex and adjusted for age, household composition, education, occupation, income, physical activity, smoking and history of dieting.

**Results:**

In women, preparation from scratch was prospectively associated with a decreased risk of obesity over the 5-year follow-up (OR = 1.32 (1.08; 2.32)) after adjustment. After including dietary mediating factors, the association between preparation from scratch and obesity risk in women did not remain significant (*P* = 0.08). This association appeared to be partly mediated by dietary factors with a difference of 59% of the estimate, in the group with the low score, between the adjusted model and those with mediators (OR = 1.13 (0.71; 1.77)). Regarding 5-year relative weight change, after adjustment for confounding factors, all associations between indicators of food preparation behaviors and weight change became non significant.

**Conclusions:**

In the context from reduced time spent preparing meals that could have an impact on dietary quality and health in industrialized countries, our prospective study does not show effect of food preparation behaviors on 5-year relative weight change and obesity risk, except for preparation from scratch on obesity risk in women. Our study provides useful information about the long term implications of food preparation behaviors on health and should be corroborated by future studies, particularly on the effect of food preparation behaviors on chronic diseases such as incident diabetes, hypertension or cardiovascular diseases, compared with other determinants.

**Trial registration:**

NCT03335644 on ClinicalTrials.gov

**Electronic supplementary material:**

The online version of this article (10.1186/s12966-018-0747-4) contains supplementary material, which is available to authorized users.

## Background

Along with the worldwide change toward increased reliance upon ultra-processed foods and increased away-from-home intake, a shift in food preparation and cooking behaviors has emerged in recent decades in industrialized countries, including France [1–7]. This change could have an important impact on dietary quality and therefore health. Previous studies have shown that lower frequency [8–10] and less time spent on meal preparation [11, 12], lower cooking skills [13–17] and lower cooking enjoyment [17–19] have been associated with unhealthier diet, such as poorer adherence to nutritional guidelines, more frequent fast-food use, higher intakes of fat and lower intakes of fruits and vegetables, fiber, folate, and vitamin A. Food preparation behaviors therefore appear to be a noteworthy determinant of dietary intake and may consequently influence weight status.

In spite of beneficial changes in dietary intake (mainly fruit and vegetables and energy intakes), intervention studies on cooking and home food preparation rarely highlight impact on adult Body Mass Index (BMI) [19–23]. According to Reicks et al. [19], these results should be interpreted with caution based on the weakness in the study design, varying duration of follow up and the lack of rigorous assessment. The few cross-sectional studies in adults have shown that time spent in food preparation was inversely related to BMI in women [2, 24] while higher consumption of foods prepared away from home was associated with higher BMI [25–28]. This may be in relation with their higher content in total fat and saturated fat compared with home prepared foods [29]. As cross-sectional design does not allow causal inferences, longitudinal studies assessing the influence of food preparation behaviors on weight change are therefore needed. To our knowledge, no study has investigated the association between food preparation behaviors and weight change with a prospective design. In addition, the contribution of dietary intake to explain the influence of food preparation behaviors on weight status has never been explored.

Food preparation behaviors are complex to define and the research literature reports on a range of indicators to assess food preparation [5, 30]. Most studies assessed food preparation behaviors by measuring time spent on food preparation [2, 11, 12, 24, 31–33] and cooking skills and knowledge [13, 14, 17–19, 30, 34–36]. Some authors were interested in enjoyment of cooking [17, 18, 31, 37, 38], others studied use of raw or fresh ingredients requiring no or minimal processing [8, 17, 38, 39], or the complexity of food preparation [10, 17]. Together, these dimensions may reflect many important elements of a food preparation behavioral pattern. However, most studies used only one dimension to capture food preparation behaviors. The conceptual model developed by Mills et al. [40] illustrates the complex, inter-linked relationships between potential determinants and outcomes of home cooking. Thus, time spent on food preparation is not only an indicator of food preparation behaviors but also a determinant of food preparation behaviors as time constraints play a major role in home cooking, particularly preparation from raw or fresh ingredients. Also, this review underlined a probable conceptual misunderstanding in that researchers conflate ‘cooking skills’ and ‘cooking’, and hence do not explicitly state and measure both concepts because they assume the two to be interchangeable [40]. In addition, Short [5] explains that the relationship between skills and knowledge and practices is not straightforward: cooks do not necessarily use convenience foods because they cannot cook, but for other reasons, including, for example, a lack of time or a lack of enjoyment, that underlies the importance of considering all these dimensions together.

The aim of our study was therefore to assess the prospective association between food preparation behaviors, captured by several dimensions, and the 5-year relative weight change and the risk of developing obesity, in a large population of French adults. In addition, we also investigated the mediating effect of dietary intake on the relationship between food preparation behaviors and weight status. Since literature has shown that gender is a much stronger determinant of food preparation behaviors than other socio-demographic variables [32–34, 38, 41], the influence of food preparation behaviors on weight change was assessed separately for men and women.

## Methods

### Study design, setting and population

We used data from the NutriNet-Santé study, a large web-based prospective observational cohort launched in France in May 2009 among volunteers from the general population of internet-using adults (> = 18 y). The cohort was designed to investigate the relationship between nutrition and health, as well as determinants of dietary behavior and nutritional status. The design, methods and rationale have been described in detail elsewhere [42]. Briefly, eligible participants were recruited by different means. At launching, a vast multimedia campaign (television, radio, national and regional newspapers, posters, and internet) called for volunteers and provided details on the study’s specific website (http://www.etude-nutrinet-sante.fr) [43]. Then, multimedia campaigns were repeated every 6 months. Further information is being maintained on a large number of websites (national institutions, city councils, private firms, web organizations). A billboard advertising campaign is regularly updated via professional channels (doctors, pharmacists, dentists, business partners, municipalities, etc.). The key message delivered in the call for volunteers was the following: “The purpose of our study is to identify nutritional risk factors or protective factors for these diseases, which is an essential step in establishing dietary recommendations to prevent the risk of disease and improve the health of the current and future generations. This is the ambitious goal of the NutriNet-Santé study and that is why researchers need you”. Aspects related to convenience of participation (ie, < 20 min each month) and confidentiality were also emphasized. In addition, a system of boosting motivation and retention was implemented [44]. In order to forge a sense of community that helps advance research, participants receive a NutriNet-Santé membership card at inclusion and a «diploma» upon completion of each follow-up year/wave. They also receive by monthly e-mail with scientific information regarding health and nutrition, and invitations to press conferences about the study results. For purposes of retention, free screening tests for cholesterol, triglycerides, and diabetes are offered to participants (the results are sent back with a special notice in case of abnormal test results). In case of an “undelivered email” problem, participants are contacted by telephone and by post to avoid drop-out. In order to be included in the cohort, participants had to complete a set of questionnaires assessing dietary intake, physical activity, anthropometry, smoking and socio-economic conditions, along with health status at baseline and each subsequent year. Additionally, each month participants were invited to complete complementary questionnaires related to the food behaviors, nutritional and health status.

### Ethics, consent and permissions

This study was conducted according to guidelines laid down in the Declaration of Helsinki, and all procedures were approved by the Institutional Review Board of the French Institute for Health and Medical Research (IRB Inserm n° 0000388FWA00005831) and the Commission Nationale Informatique et Libertés (CNIL n° 908,450 and n° 909,216). This study is registered in ClinicalTrials.gov (n° NCT03335644). Written electronic informed consent to participate in the study was obtained from all subjects.

### Data collection

This longitudinal analysis focused on participants included in the NutriNet-Santé cohort study between May 2009 and May 2011. Food preparation behaviors were assessed in May 2011. Data regarding sociodemographic, lifestyle and behavioral characteristics and dietary intake used in this analysis were also collected in 2011. Weight and height data were collected in 2011 and 5 years later, in 2016.

#### Food preparation behaviors

Based on published literature available at the time [5, 13, 17, 30, 45], food preparation behaviors were captured by several dimensions: cooking frequency, daily time spent on food preparation, preparation from scratch, cooking skills, cooking enjoyment, willingness to cook better or more frequently, and kitchen equipment. Recently, the area of measuring cooking skills and food preparation behaviors has been advanced in terms of development and validation, including new dimensions such as food self-efficacy, food attitude and the influence of non-food barriers [46, 47].

Face validity was assessed by experts and subjects. A team of multidisciplinary researchers (nutritionists, dieticians, economists and sociologists) developed the questionnaire. They evaluated whether the items were relevant to assess the measured concept, and only that concept, and whether they constituted a representative sample of a set of items describing the concept. Experts also evaluated the quality of the visible features of the items: length, items’ wording, and categories of response. Acceptability and feasibility were also measured in 100 subjects using specific questions on the perceived complexity and difficulty of filling in the questionnaire and whether the questionnaire was too long or any items were redundant, using a 4-point Likert scale from “I strongly disagree” to “I strongly agree”. For 97% of the sub-sample who assessed the feasibility of the questionnaire, the questionnaire was not difficult; only 1% found it difficult. For 93% of the participants, the questionnaire was not annoying; only 6% found it annoying. Finally, 82% thought the questionnaire was not too long but 10% found it too long. Test–retest reliability (repeatability) on variables was assessed in 22 subjects who filled out the questionnaire twice at a two-week interval, by computing the prevalence- and bias adjusted kappa coefficient (PABAK) for each item of the questionnaire [48]. Repeatability was considered satisfactory for a PABAK higher than 0.40, with an interpretation similar to Cohen’s kappa coefficient. Only four items had a PABAK < 0.40 (Additional file 1: Table S1). PABAK for other items was between 0.35 and 0.67.

##### Frequency and time for meal preparation

Participants were asked who the main cook in the household was and how often they prepared meals during a typical week, including preparation of a cold dish or reheating a prepared dish (two or more times per day, once a day, several times a week but not every day, once a week, less than once a week, never). If participants answered never, no further questions regarding food preparation behaviors were asked. Otherwise, participants were asked how much time (in minutes) they usually spent preparing meals, including cooking time. To assess average daily time preparing food, we multiplied the duration for meal preparation by the frequency and divided by 7.

##### Preparation from scratch

The definition of “preparation from scratch” generally suggest cooking with raw, fresh or minimally processed foods and without the use of ultra-processed foods for which consumption is known to be associated with lower nutritional quality and higher risks of obesity and chronic diseases [49]. To assess preparation from scratch participants were asked about their use of foods (fruit, vegetable, fish and meat) according to their processing level. In addition, among unprocessed meat and fish, we have distinguished forms of unprocessed meat or fish according to the investment and the techniques to prepare them (for instance whole fish not cleaned out vs. fish fillets, sliced, pavers or steaks). Use of fruit or vegetables with no or minimal processing was assessed with the questions “Among the following fruits/vegetables, which ones do you use unpeeled, uncut, unprocessed?”. The ten groups of vegetables proposed covered the foods more consumed in France categorized by groups usually purchased by French consumers (Additional file 1: Table S2) [50]. Use of unprocessed fruit and use of unprocessed ‘tomato, pepper, eggplant’, ‘cucumber, zucchini’, ‘garlic, onions, shallots’ and potatoes were excluded from the score calculation as more than 95% of the participants used these items. When a participant self-reported the use of an unpeeled, uncut, unprocessed vegetable group, 1 point was allocated (Additional file 1: Table S2). For fish, participants had to determine what forms of fish they usually use (whole fish not cleaned out, whole fish cleaned out, fish fillets, sliced, pavers or steaks, breaded fish). Participants also had to assess what forms of meat they usually use (chunky uncut pieces, whole poultry not cleaned out, whole poultry cleaned out, cut poultry or meat, ready to cook poultry or meat such as ultra-processed meat and nuggets). Participants were allowed to select several types of fish and meat. When a participant self-reported the use of whole fish, 2 points were allocated (Additional file 1: Table S2). When a participant self-reported the use of breaded fish and the use of other forms, 1 point was allocated, whereas when participants only reported breaded fish or no other form of fish, no points were allocated. When a participant self-reported the use of chunky uncut pieces, whole poultry not cleaned out, whole poultry cleaned out, cut poultry or meat (even if participant also used ready to cook poultry or meat), 1 point was allocated whereas, when participants only reported ready to cook poultry or meat such as ultra-processed meat and nuggets, no points were allocated. A score of preparation from scratch was calculated from 0 to 12 points according to answers regarding use of raw vegetables, forms of fish and meat used (Additional file 1: Table S2).

As Pearson correlations are not adapted to non-continuous variables and tend to underestimate the relationships between ordinal variables, polychoric correlations were used in this analysis [51]. Internal consistency for preparation from scratch was therefore estimated with the ordinal alpha coefficient [52], which is more accurate in estimating alphas for measurements involving ordinal variables [52]. Although it is calculated using polychoric correlations, it is conceptually equivalent to Cronbach’s alpha and has a similar interpretation, i.e. a value higher than 0.70 is considered adequate [53]. Ordinal alpha value was 0.72 for the dimension assessing preparation from scratch, indicating adequate internal consistency (Additional file 1: Table S3). Then, polychoric correlations between the items and their respective dimension corrected for overlap (i.e., the modified subscale after removal of the studied item) were also computed. The aim of this analysis was to verify that items were substantially correlated with their assigned dimension. A polychoric correlation ≥0.40, corrected for overlap, is considered adequate. For this dimension, corrected item total polychoric correlations were all above 0.40 (Additional file 1: Table S3).

##### Cooking skills

Participants were also asked to assess their cooking skills regarding 7 dishes, 8 pastries and sweets, 7 sauces and 4 cooking techniques (Additional file 1: Table S2). A large range of foods was used, both including generic items previously used in studies [9, 27, 54] and specific dishes/pastries frequently consumed in France [50], to assess the ability to cook the dishes rather than ability to choose the healthier dishes and to therefore avoid desirability bias to self-report preparation of healthy vs. unhealthy dishes/pastries. Skills to cook dishes and pastries were evaluated using two types of questions, according to the dishes/pastries and sweets. For instance, to the question ‘Do you know how to make pancakes or waffles?’ participants could answer “Yes, I know”, “Yes, I know but only with ready for use pancake batter”, “No, I don’t know” and “I’ve never tried”. Individuals tend to adopt a more time-efficient food preparation behavior that influence their cooking skills [40]. To reflect this behavior in assessment of cooking skills, when a participant reported making the more complex variant of the dish/pastry/sweet, i.e. entirely homemade, without ready for use ingredients, 2 points were allocated whereas, when participants reported preparing the variant with ready for use ingredients, 1 point was allocated. When ready for use preparation does not exist for a dish/pastry, participants could answer “Yes, I know”, “No, I don’t know” and “I’ve never tried”. Some dishes such as omelet and vegetable soup were not included in the score as more than 90% of the participants declared making these dishes. The same answers were proposed to evaluate cooking techniques. When a participant answered “Yes, I know”, 1 point was allocated. Skills regarding sauces were evaluated using the question “Among the following sauces, which ones do you know how to prepare?”. Points were allocated according to the skill complexity of the sauces and the number of sauces reported: 4 points for hollandaise sauce or sauce by reduction, 3 points for 3 or 4 simple sauces (mayonnaise, garlic butter, béchamel, tomato sauce), 2 points for 2 simple sauces, 1 point for 1 simple sauce, no point for salad dressing (Additional file 1: Table S2). A score of cooking skills was calculated from 0 to 41 points based on skills to make dishes, pastries, sauces and cooking techniques (Additional file 1: Table S2). Internal consistency for cooking skills was also estimated with the ordinal alpha coefficient [52] for which the value found was 0.84, indicating adequate internal consistency (Additional file 1: Table S3). For cooking skills, corrected item total polychoric correlations were all above 0.40 (Additional file 1: Table S3).

##### Kitchen equipment

Kitchen equipment was assessed by the question “Is your kitchen equipped with the following utensils and appliances?”. Using data from French statistics on income and living conditions [55], seven utensils and appliances were proposed: pressure cooker, zester, baking pan, measuring cup, food processor, gas oven or electric oven. When a participant reported possessing kitchen equipment, 2 points were allocated, except for common equipment such as gas oven or electric furnace for which 1 point was allocated. Kitchen equipment was transformed into a score from 0 to 11 points (Additional file 1: Table S2).

##### Enjoyment for food preparation and willingness to cook better and more frequently

Participants were asked to assess their enjoyment of food preparation using the question: “Do you enjoy cooking?” (Yes, including daily meal preparation; Yes, but not daily meal preparation; No). They were also asked to assess their willingness to improve their cooking skills, whatever the skill level, and to cook more frequently using the following questions: “Do you wish to cook better?” (Yes/No), “Do you wish to cook more often?” (Yes/No).

#### Assessment of weight and BMI

Height and weight data were collected at baseline and each year thereafter by a self-administered anthropometric questionnaire [56]. BMI (kg/m^2^) was calculated as the ratio of weight to the square of height. Obesity was defined as body mass index greater than or equal to 30 kg/m^2^ in accordance with WHO reference values [57]. The 5-year relative weight change was computed as: ((5y weight – baseline weight)/(baseline weight)) × 100 and was expressed as a percentage of the baseline weight.

#### Assessment of dietary intake

At enrolment and each year thereafter, participants were invited to provide three random 24 h dietary records during a two-week period (1 weekend day and 2 weekdays) [58]. Data used in this analysis were collected using 24 h dietary records at the same time as data of food preparation behaviors in 2011. Participants were invited to declare every beverage and food consumed during the day. They estimated portion sizes using validated photographs or usual containers [59], representing more than 250 foods (corresponding to 1000 generic foods) served in seven different portion sizes. The values for energy were estimated using published nutrient databases [60] and completed for recent market foods and recipes. Under-reporters were excluded using the method proposed by Black [61]. The accuracy of web-based 24 h dietary records has been assessed by comparing to interviews by trained dietitians [58] and also against 24 h urinary biomarkers [62, 63].

#### Sociodemographic, lifestyle and behavioral characteristics

Potential confounding factors of the relationship between food preparation behaviors and the 5-year relative weight change previously identified [64] were collected using web-based questionnaires at the same time as data of food preparation behaviors: age (years), household composition (single, couple without a child, couple with ≥1 child, and household without a child but with ≥3 adults), education (primary education, secondary education, undergraduate, corresponding to up to 3 years after the high school, and post-graduate corresponding to more than 3 years after the high school diploma), occupation (manual and office worker, intermediate profession, managerial staff, self-employed and never-employed), monthly household income (< 1200 €, 1200–1800 €, 1800–2700 € and > 2700 €, plus a category for individuals who were unwilling to answer), smoking status (never, former or current smoker), history of dieting (never, former, or current dieter) and physical activity level using the French version of the International Physical Activity Questionnaire [65] (low, moderate or high).

### Statistics

**Fig. 1:**
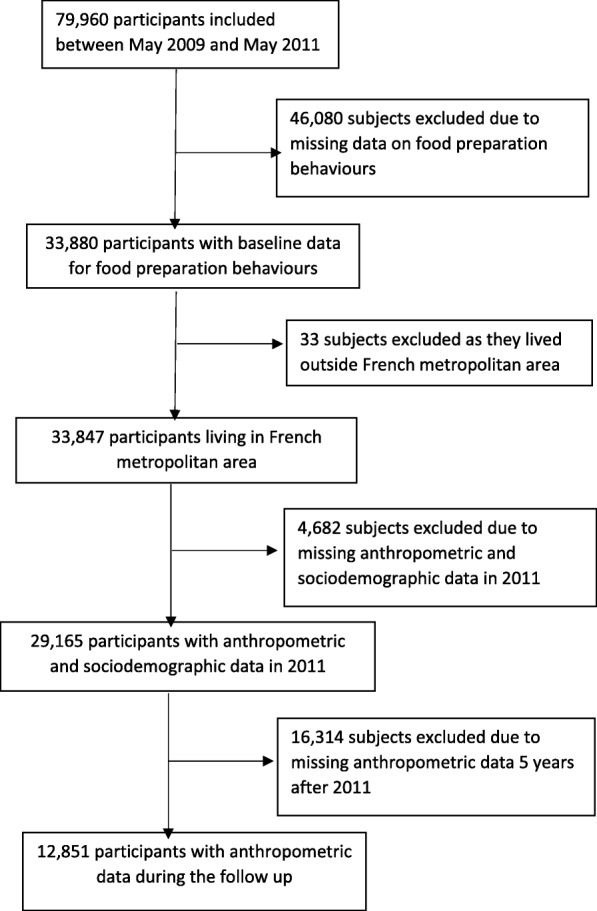
Flowchart

Our analysis focused on participants included in the NutriNet-Santé study between May 2009 and May 2011, living in the French metropolitan area, who had self-reported height and weight data at baseline (2011) and 5 years later, who had completed three 24 h dietary records at baseline, who were not energy under-reporters and who had no missing data for socioeconomic and demographic factors (Fig. 1). The first set of analyses assessed the association between food preparation behaviors and 5-year relative weight change in 12,851 subjects whatever their BMI at baseline. The second set of analyses was restricted to non-obese subjects at baseline (*n* = 11,502) and assessed the 5-year risk of becoming obese. Regarding frequency for meal preparation, we performed analyses on the whole sample, including non-cooks (those who cook less than once a week or never). Then, analyses for the other indicators of food preparation were only conducted among regular and occasional cooks.

Time for food preparation and each score (preparation from scratch, cooking skills and kitchen equipment) were transformed into tertiles. All analyses were performed separately for men and women, since almost all interactions between gender and the food preparation indicators were significant (P interaction < 0.10). Descriptive comparisons between sexes were performed using Student’s t test and the Chi-square test.

#### 5-year relative weight change

The predictive value of each indicator of food preparation behaviors on the 5-year relative weight change was assessed by multivariate linear regressions. The SAS procedure used was PROC GLM, where the dependent variable was weight change as percentage of the baseline weight, and the independent variables were indicators of food preparation behaviors and all covariates. For each indicator, two different models were used. The first models were performed to study the independent effect of each indicator of food preparation behaviors on weight change. The second model was adjusted for age, household composition, education, household income, occupation, physical activity level, dieting to lose weight and smoking status.

#### Obesity risk

In a second set of analyses, we estimated the odds ratios (OR) and 95% confidence intervals (CI) of becoming obese after 5 years of follow-up, after exclusion of 1348 obese subjects at baseline, using multivariate logistic regressions, stratified by sex. The SAS procedure used was PROC LOGISTIC, where the dependent dichotomous variable was ‘being obese/ not being obese at the end of follow-up’. Two models were used: model 1 was a base model only including the indicator of food preparation behaviors; model 2 also included age, household composition, education, household income, occupation, physical activity level, dieting to lose weight and smoking status.

#### Mediation analysis

Analyses on the mediating effect of dietary intake on the relationship between indicator of food preparation behaviors and 5-year relative weight change or obesity risk were only performed when statistically significant association between the indicator and weight change or obesity risk in adjusted models was observed. To assess the mediating effect of dietary intake, we selected food groups for which the intake was associated with 5-year relative weight change or obesity risk as well as the indicator of food preparation behaviors using linear or logistic regression models, as appropriate (*P* ≤ 0.01). Then, we fitted models for weight change or obesity risk that included indicator of food preparation behaviors, confounders and, successively, the dietary factors previously selected. The magnitude of the mediating effect was assessed by the percentage change in the ORs or the β of the different groups computed as [(OR/β base model – OR/β base model + mediator) / (OR/β base model − 1)] × 100. We applied a quantitative criterion to see whether dietary intake had a mediating effect. To do so, a 10% reduction threshold was used to consider dietary intake as a mediating factor.

To optimize the robustness of the statistical tests, we performed sensitivity analyses. First, sensitivity analyses were performed only in individuals who reported being the main cook in the household for all dimensions of food preparation behaviors. Second, we redefined the outcome as the risk of becoming overweight in order to overcome the potential misclassification bias. For these analyses, we used an identical approach as described above. A *P*-value < 0.05 was initially considered statistically significant. Data management and statistical analyses were performed using SAS (version 9.1; SAS Institute, Inc., Cary, NC, USA).

## Results

### Participation

A total of 33,884 individuals had completed the complementary questionnaire measuring food preparation behaviors, i.e. 42% of participants included between May 2009 and May 2011 in the Nutrinet-Santé cohort who were invited to fill in the questionnaire. This low participation rate was due to the optional nature of the questionnaire administration.

### Baseline characteristics of participants

A total of 9222 women and 3629 men were included in this analysis (Fig. 1). Comparisons between excluded and included participants in the analysis showed that the percentages of young participants (18–24 y.), individuals living with at least one child, never-employed persons, employees/manual workers and persons with income < 1200 euros were lower in the final sample used for analyses (all *P* values < 0.0001; data not shown). Difference was observed between the overall sample and those with missing data who were excluded (Additional file 1: Table S4). The percentages of women, young adults, subjects with low income, those with low physical activity level, and current dieter were higher in excluded subjects, compared to the overall sample (Additional file 1: Table S4). The energy intake of excluded subjects was lower while their intake of alcoholic beverages was higher, compared to the overall sample.

Overall, weight evidenced a change of + 1.2% (6.9) in women and + 0.6% (5.8) in men. At baseline, men were twice as likely as women to be overweight while prevalence of obesity was equivalent in both genders (Table 1). Men were slightly less likely to become obese by the 5-year follow-up (54 cases (1.7%) in men vs 154 cases (1.9%) in women). Percentages of young adults, those who lived with at least one child, participants with an undergraduate educational level, manual workers and office workers, never-employed and those in the lowest income class were higher among women than among men (Table 1).

**Table 1:** Demographic and socioeconomic characteristics of the sample at baseline and 5-year relative weight change^a^ (*n* = 9222 women and *n* = 3629 men)

	Women % or mean (SD)	Men % or mean (SD)	*P*-value^2^
5-year weight change (%)	1.2 (6.9)	0.6 (5.8)	< 0.0001
BMI classes			< 0.0001
Normal (< 25 kg/m2)	73.1	54.2	
Overweight (< 30- ≥ 25 kg/m2)	18.6	36.4	
Obese (≥30 kg/m2)	8.3	9.4	
Education			< 0.0001
Primary	2.9	3.9	
Secondary	32.9	35.7	
Under-graduate	32.4	22.2	
Post-graduate	31.8	38.2	
Occupation			< 0.0001
Never employed	3.0	1.1	
Self-employed	2.5	4.2	
Manual worker, office worker	30.4	14.3	
Intermediate profession	31.5	24.3	
Managerial staff	32.6	56.1	
Monthly household income per household unit			< 0.0001
Unwilling to answer	9.7	4.7	
< 1200 euros	13.0	9.5	
1200–1800 euros	39.7	36.4	
1801–2700 euros	9.6	11.8	
> 2700 euros	28.0	37.6	
Age			< 0.0001
18–24 years	3.0	1.2	
25–34 years	14.0	7.6	
35–54 years	44.3	31.1	
> 55 years	38.7	60.1	
Household composition			< 0.0001
Single	17.7	13.5	
Couple without child	39.9	53.6	
Couple with ≥one child	31.9	24.0	
Household without child and with ≥3 adults	10.5	8.9	
Physical activity level			< 0.0001
Low	31.9	45.3	
Moderate	45.7	38.2	
High	22.4	16.5	
Smoking status			< 0.0001
Never-smoker	53.6	41.2	
Former smoker	34.3	48.5	
Current smoker	12.1	10.3	
Dieting to lose weight			< 0.0001
Never dieter	17.9	32.1	
Former dieter	70.6	61.5	
Current dieter	11.5	6.4	

^2^*P*-value represented the overall significance of each variable (Type 3 analysis of effects)

^a^5-year relative weight change between 2011 and 2016

Percentages of regular cooks, individuals who enjoy cooking including daily meal preparation and those who wished to cook better were higher in women than in men while the percentages of non- and occasional cooks were lower (Table 2). Women spent more time for meal preparation and had higher scores of cooking skills and kitchen equipment than men (Table 2).

**Table 2:** Food preparation behaviors in men and women^a^

All subjects	Women % or mean (SD)	Men % or mean (SD)	*P*-value^2^
Frequency of meal preparation	*n* = 9222	*n* = 3629	< 0.0001
Non-cook (less than once/week or never)	1.3	19.8	
Occasional cook (less than once/day but at least once/week)	17.3	35.6	
Regular cook (once or more times/day)	81.4	44.6	
Occasional and regular cooks only	*n* = 9097	*n* = 2911	
Time for meal preparation (min/day)	40.2 (23.3)	28.4 (22.0)	< 0.0001
Cooking skills (0–41 point score)	21.9 (5.3)	18.4 (7.4)	< 0.0001
Preparation from scratch (0–12 point score)	7.7 (2.3)	7.4 (2.5)	0.08
Kitchen equipment (0–11 point score)	8.2 (2.0)	7.2 (2.6)	0.002
Enjoy cooking			0.0002
Yes, including daily meal preparation	70.3	68.7	
Yes, but not daily meal preparation	18.8	17.6	
No	10.9	13.7	
Willingness to cook better			0.006
Yes	65.0	62.1	
No	35.0	37.9	
Willingness to cook more frequently			0.96
Yes	28.1	28.2	
No	71.9	71.8	

^2^*P*-value represented the overall significance of each variable (Type 3 analysis of effects)

^a^time spent for food preparation, preparation from scratch cooking skills, kitchen equipment, enjoy cooking, willingness to cook better and willingness to cook more frequently were only assessed in occasional and regular cooks (*n* = 12,008)

### Association between food preparation behaviors and 5-year relative weight change

In base model in women, positive 5-year relative weight change slightly decreased linearly from the lowest to the upper tertiles of scores for cooking skills (− 0.47%, for instance a decrease of 320 g for a subject with a weight of 70 kg at baseline) and preparation from scratch (− 0.82%, linear trend *P* < 0.05) (Table 3). Individuals who wished to cook better (+ 0.55% in women; + 0.68% in men) and women who wished to cook more frequently (+ 0.60%,) slightly gained more weight than those who did not. In the fully adjusted models, all associations became non significant (Table 3).

**Table 3:** Associations between food preparation behaviors and 5-year relative weight change (*n* = 12,851)^a^

	Base model^b^						Adjusted model^c^					
	Women			Men			Women			Men		
	Mean^d^	SEM	*P*-value^5^	Mean^d^	SEM	*P*-value^5^	Mean^d^	SEM	*P*-value^5^	Mean^d^	SEM	*P*-value^5^
Frequency of meal preparation												
Non-cook (less than once/week or never)	0.90	0.63	0.12	0.46	0.22	0.67	1.28	0.88	0.29	1.15	0.49	0.60
Occasional cook (less than once/day but at least once/week)	1.12	0.08		0.63	0.14		1.34	0.26		1.13	0.43	
Regular cook (once or more times/day)	1.51	0.17		0.46	0.16		1.73	0.33		0.88	0.45	
Time for meal preparation												
Low (2–22 min/day in women; 2–13 min/day in men)	1.35	0.13	0.21	0.52	0.18	0.96	0.99	0.51	0.53	1.34	0.29	0.90
Medium (22–38 min/day in women; 13–30 min/day in men)	1.17	0.13		0.55	0.23		0.99	0.55		1.37	0.28	
High (39–106 min/day in women; 30–50 min/day in men)	1.04	0.13		0.59	0.18		1.33	0.50		1.45	0.29	
Cooking skills			**0.03**			0.81			0.26			0.76
Low	**1.44**	**0.13**		0.56	0.19		1.15	0.29		1.06	0.53	
Medium	**1.18**	**0.12**		0.64	0.19		1.50	0.28		1.09	0.52	
High	**0.97**	**0.13**		0.46	0.19		1.47	0.28		1.31	0.52	
Preparation from scratch			**< 0.0001**			0.05			0.81			0.72
Low	**1.69**	**0.14**		0.93	0.20		1.45	0.30		1.26	0.51	
Medium	**1.16**	**0.13**		0.46	0.20		1.45	0.29		0.96	0.52	
High	**0.87**	**0.11**		0.30	0.18		1.32	0.28		1.16	0.51	
Kitchen equipment			0.05			0.70			0.90			0.42
Low	1.42	0.12		0.71	0.22		1.40	0.28		1.17	0.54	
Medium	1.05	0.10		0.51	0.22		1.37	0.18		0.83	0.52	
High	1.06	0.20		0.49	0.16		1.40	0.34		1.30	0.50	
Enjoyment for food preparation			0.62			0.57			0.94			0.68
No	1.16	0.22		0.36	0.30		1.31	0.37		0.93	0.59	
Yes, but not daily meal preparation	1.33	0.17		0.40	0.26		1.40	0.32		0.98	0.58	
Yes, including daily meal preparation	1.16	0.09		0.63	0.13		1.40	0.26		1.22	0.48	
Willingness to cook better			**0.0003**			**0.003**			0.33			0.10
No	**0.83**	**0.12**		**0.13**	**0.18**		1.26	0.23		0.83	0.33	
Yes	**1.38**	**0.09**		**0.81**	**0.14**		1.46	0.21		1.33	0.32	
Willingness to cook more frequently			**0.0002**			0.08			0.14			0.73
No	**1.02**	**0.09**		0.43	0.13		1.30	0.26		1.11	0.32	
Yes	**1.62**	**0.14**		0.86	0.21		1.63	0.30		1.23	0.30	

^5^*P*-value represented the overall significance of each variable (Type 3 analysis of effects)

^a^time spent for food preparation, preparation from scratch cooking skills, kitchen equipment, enjoy cooking, willingness to cook better, willingness to cook more frequently were only assessed in occasional and regular cooks (*n* = 12,008)

^b^base model only included only one indicator of food preparation behaviors

^c^Adjusted model: base model + adjustment for age, household composition, education, household income, occupation, physical activity level, dieting to lose weight and smoking status

^d^Values are mean weight change expressed as the percentage of baseline weight (%)

In bold, results considered as significant, i.e. with a *p*-value < 0.05

### Association between food preparation behaviors and obesity risk

The only significant association between indicators of food preparation behaviors and obesity risk concerns preparation from scratch in women (Table 4). In base model, women with low score for preparation from scratch were more likely to become obese by the 5-year follow-up than women with high score. This association remained significant after adjustment for sociodemographic, lifestyle and behavioral factors and was not observed in men.

**Table 4:** Associations between food preparation behaviors and risk of becoming obese after 5 years of follow-up (*n* = 11,502)^a^

	Base model^b^						Adjusted model^c^					
	Women			Men			Women			Men		
	OR	95%CI^d^	*P*-value^5^	OR	95%CI^d^	*P*-value^5^	OR	95%CI^d^	*P*-value^5^	OR	95%CI^d^	*P*-value^5^
Frequency of meal preparation			0.72			0.67			0.70			0.13
Non-cook (less than once/week or never)	1.80	0.89–3.65		1.80	0.89–3.65		1.00	0.12–4.13		2.65	0.93–7.94	
Occasional cook (less than once/day but at least once/week)	1.57	0.84–2.94		1.57	0.84–2.94		0.81	0.44–1.49		2.41	0.90–6.42	
Regular cook (once or more times/day)	1.00			1.00			1.00			1.00		
Time for meal preparation			0.81			0.94			0.93			0.24
Low (2–22 min/day in women; 2–13 min/day in men)	0.94	0.64–1.39		1.13	0.55–2.29		1.11	0.40–1.95		2.31	0.70–2.59	
Medium (22–38 min/day in women; 13–30 min/day in men)	0.88	0.59–1.31		1.03	0.45–2.36		1.06	0.61–1.82		2.82	0.79–1.01	
High (39–106 min/day in women; 30–50 min/day in men)	1.00			1.00			1.00			1.00		
Cooking skills			0.72			0.08			0.49			0.13
Low	0.97	0.65–1.43		0.78	0.39–1.55		0.98	0.45–1.36		1.25	0.45–3.47	
Medium	0.86	0.58–1.27		0.37	0.15–0.89		0.74	0.44–1.25		0.25	0.05–1.24	
High	1.00			1.00			1.00			1.00		
Preparation from scratch			**0.008**			0.98			**0.02**			0.67
Low	**1.69**	**1.11–2.56**		0.98	0.46–2.07		**1.32**	**1.08–2.32**		1.36	0.44–4.18	
Medium	1.02	0.68–1.54		0.93	0.43–2.00		0.95	0.56–1.64		0.75	0.22–2.65	
High	1.00			1.00			1.00			1.00		
Kitchen equipment			0.25			0.55			0.56			0.31
Low	1.53	0.88–2.66		0.81	0.38–1.72		1.49	0.70–3.17		2.00	0.68–2.90	
Medium	1.25	0.72–2.16		0.64	0.29–1.45		1.30	0.62–2.71		0.79	0.20–3.16	
High	1.00			1.00			1.00			1.00		
Enjoyment for food preparation			0.17			0.85			0.62			0.55
Yes, including daily meal preparation	1.31	0.75–2.28		1.20	0.46–3.11		1.43	0.60–3.67		1.33	0.28–2.33	
Yes, but not daily meal preparation	0.86	0.44–1.71		0.97	0.29–3.20		1.49	0.67–3.29		0.69	0.18–2.67	
No	1.00			1.00			1.00			1.00		
Willingness to cook better			0.12			0.58			0.19			0.67
Yes	1.32	0.92–1.87		0.84	0.45–1.57		1.38	0.84–2.34		0.81	0.30–2.16	
No	1.00			1.00			1.00			1.00		
Willingness to cook more frequently			0.08			0.96			0.01			0.54
Yes	1.35	0.97–1.89		0.98	0.49–1.98		1.83	1.15–2.93		1.43	0.45–4.51	
No	1.00			1.00			1.00			1.00		

^5^*P*-value represented the overall significance of each variable (Type 3 analysis of effects)

^a^time spent for food preparation, preparation from scratch cooking skills, kitchen equipment, enjoy cooking, willingness to cook better, willingness to cook more frequently were only assessed in occasional and regular cooks (*n* = 10,775)

^b^base model only included the corresponding indicator of food preparation behaviors

^c^Adjusted model: base model + adjustment for age, household composition, education, household income, occupation, physical activity level, dieting to lose weight and smoking status

^d^95% Confidence Interval

In bold, results considered as significant, i.e. with a *p*-value < 0.05

Regarding mediation analysis, women with lower score for preparation from scratch had lower intake of fruits and vegetables (mean intake: 376.8 g/d (230.3) for low score (LOW) and 487.9 g/d (231.2) for medium score (MED) and. 528.9 g/d (217.9) for high score (HIGH); *P* < 0.0001), whole-grain products (LOW: 30.2 g/d (45.1), MED: 35.8 g/d (43.9), HIGH: 39.0 g/d (46.1); P < 0.0001), oils (LOW:7.8 g/d (7.3), MED: 8.9 g/d (7.7), HIGH: 9.9 g/d (8.5), P < 0.0001) and alcoholic beverages (LOW:68.4 g/d (105.5), MED: 74.9 g/d (110.8), HIGH: 93.1 g/d (116.9); P < 0.0001) whereas they had higher intake of fatty sweet foods (LOW: 23.5 g/d (30.4), MED: 20.5 g/d (25.9), HIGH: 16.9 g/d (23.6); *P* < 0.0001) compared with participants with higher scores. In addition, new obese subjects had lower intake of fruits and vegetables (mean intake: 375.8 g/d (282.1) vs. 458.3 g/d (291.8); P < 0.0001), whole-grain products (27.1 g/d (36.6) vs. 35.8 g/d (45.3); *P* = 0.02), oils (7.8 g/d (7.1) vs. 9.9 g/d (8.8); *P* < 0.0001) and higher intake of fatty sweet foods (11.0 g/d (20.5) vs. 12.0 g/d (22.1); *P* = 0.001) and alcoholic beverages (85.9 g/d (144.6) vs. 80.6 g/d (111.8); *P* < 0.0001) compared to those that remained non-obese.

After including dietary mediating factors, the association between preparation from scratch and obesity risk in women did not remain significant (*P* = 0.08). This association appeared to be partly mediated by dietary factors with a difference of 81% of the estimate in the group with the low score, between the base model and those with mediators (OR = 1.13 (0.71; 1.77)) and 59% of the estimate between the adjusted model and those with mediators.

Sensitivity analysis that excluded individuals who were not the main cook in the household did not change the results (data not shown). Then, when the outcome was the risk of overweight, all associations between indicators of food preparation behaviors and risk to become overweight were non-significant (data not shown). The addition of overweight status as confounding factor in the adjusted model that assessed associations between food preparation behaviors and risk of becoming obese after 5 years of follow-up very slightly changed the ORs. In unadjusted model for baseline overweight status in women, preparation from scratch was associated with a decreased risk of obesity over the 5-year follow-up (OR low score vs. high =1.32 (1.08; 2.32)). After the inclusion of this variable in the adjusted model for women, the ORs of preparation from scratch became: OR low score vs. high = 1.31 (1.07; 2.46).

## Discussion

To the best of our knowledge, this is the first published study to investigate prospective associations between food preparation behaviors and 5-year relative weight change. We highlighted that preparation from scratch was prospectively associated with a slight decreased risk of obesity in women and dietary intake appeared to substantially explain this relationship. Results have also shown that there was no significant association between other indicators of food preparation behaviors and 5-year relative weight change after adjustment for sociodemographic, lifestyle and behavioral factors, suggesting a poor impact of food preparation behaviors (as measured in this study) on weight change over time.

No study has explored the influence of use of fresh or minimally processed foods on weight change. However, our finding is in line with previous works which reported higher obesity prevalence in subjects with greater household availability and higher intake of ready-to-eat or ready-to-heat foods known as ultra-processed foods, compared with those who mainly used and consumed fresh or minimally processed foods [66–68]. Indeed, ready-to-eat foods are more energy-dense and stimulate overconsumption by their hyper-palatability, large portion sizes, convenience, than the combination of foods and ingredients made into freshly prepared meals [66]. Compared to those with high score of preparation from scratch, women with lower score had higher intake of ultra-processed fatty-sweet products and lower intake of no or minimally processed foods which are more likely to be prepared from scratch, such as fruits and vegetables and whole-grain products. Such dietary behaviors represent a nutritional difference between women with high score and those with low score of preparation from scratch (e.g. + 152 g/day in intake of fruits and vegetables) that could have long-term consequences on body weight [69]. Unlike for the risk of obesity, association between preparation from scratch and 5-year relative weight change in women was not significant after adjustment for sociodemographic and lifestyle factors, due to the confounding effects of age, education, smoking status and history of dieting. Indeed, these factors were positively associated with score of preparation from scratch and inversely associated with 5-year relative weight change (lower weight change with increased age and education and among never-smokers and never-dieters) while they were not significantly associated with obesity risk.

Women with greater cooking skills and women who did not wish to cook better or more frequently gained less weight than those with low skills and those with willingness to cook better and more frequently, but this association did not remain significant after adjustment for confounding factors. In particular, we observed a strong confounding effect of age in these relationships, when adding this variable in the model. Descriptive analysis showed that older women gained less weight over 5 years than younger participants (0.15 (5.65) in subjects > 65y. vs. 2.68 (7.78) in subjects < 25 years; *P* < 0.0001) that may be explained by physiological changes [70]. Consistently with previous studies [14, 34, 71], the group with high score of cooking skills was mostly constituted of women > 65 years (44% of the 3rd tertile while subjects < 25 y. were 12%). The potential beneficial effect of cooking skills on weight gain may therefore be due to generational influence. Compared with women of older generations, young generations had less acquired cooking skills (score of cooking skills in women < 25 years: 4.0 (1.1) vs. women > 65 years: 5.2 (1.3), *P* < 0.0001). This may be due to a decline in the intergenerational transmission of basic cooking skills at home [72]. Also, young women may assume less the role of the main food provider for the family and also due to increased exposure to convenience foods [73]. This last may decrease the importance of homemade traditions and the use of raw and unprocessed ingredients (score of preparation from scratch in women < 25 years: 4.1 (1.0) vs. women > 65 years: 6.5 (1.4), *P* < 0.0001); there may also be an age effect, those over 65 being retired, and less subject to time pressure than the younger [74]. Regarding willingness to cook better and more frequently, less than 2% of 18–24 y. subjects did not wish to cook better or more frequently versus more than half in old subjects (> 55 y), as they already spent more time preparing meals. Indeed, after adjustment for time spent preparing food, the associations between willingness to cook better and more frequently and 5-year relative weight change did not remain significant (results not shown).

Our findings showing no significant prospective association between frequency or time for food preparation and weight status over time, either in univariate and multivariate analyses, did not reinforce results from cross-sectional studies highlighting inverse relationships with BMI [2, 24, 75]. Such discrepancies may be explained by difference in study design. The reverse causality cannot be excluded in cross-sectional studies whereas the prospective design with the 5 years of follow-up allows us to explore the inference of causality between food preparation behaviors and weight outcomes. This suggests a large nutritional heterogeneity of home-prepared meals for the same time spent and beneficial effect of food preparation behaviors on weight status may be rather due to the choice to home-cook high dietary quality meals than the time invested for preparation.

The non-significant associations between food preparation behaviors and weight status over 5 years in men (except for willingness to cook better in base model) were concordant with previous studies showing significant associations between indicators of food preparation (time for food preparation, complexity of food preparation, cooking skills) and diet quality or BMI in women rather than in men [2, 14, 24, 76]. A large body of literature studied the relationship between gender and food preparation behaviors. Women were more likely than men to be involved with cooking, spend time to cook and feel confident cooking [40]. A previous French study also showed that men perceived food preparation as both a chore and a leisure activity while women perceived food preparation as a way to eat healthy [77]. Unlike women, male cooks may prepare more frequently traditional dishes, rich in fats that not healthier than ready-to-eat meals and consequently food preparation behaviors in men may be not a lever for healthier dietary intake and better weight status. In addition, absence of such association in men may result of the low proportion of main cooks among male individuals (14% vs 85% in women). Our findings indicated that interventions targeted at women may have greatest impact on health, but interventions to engage men further in food preparation behaviors are an opportunity to address gender issues and the sharing of household activities.

The strengths of our study pertained to its prospective design, its large sample size, and the quantitative assessment of dietary intake. Nevertheless, some limitations should be acknowledged. First, caution is needed regarding the extrapolation of these results to the entire French population since this study included adults involved in a long-term cohort study investigating the association between nutrition and health, with overall more health-conscious behaviors and higher socio-professional and educational levels [44, 78, 79]. In addition, subjects included in the analysis sample were less likely to be current dieters and were more physically active than the overall sample. Thus, weight changes may have been underestimated in this study compared with the general population, which may have weakened the associations. In addition, the web-based design might not increase, but possibly even mitigate recruitment biases [80]. Indeed, a previous work regarding participants in our cohort showed that the exclusive use of the Internet for data collection and follow-up may help to increase the proportion of population groups which are often underrepresented in volunteer cohorts such as men and older subjects [44]. In addition, previous work showed a great geographic and socio-demographic diversity in participants at baseline in the NutriNet-Santé study, which showed resemblance in terms of age and income distribution with the French general population [78].

As food preparation behaviors and confounding factors have been assessed at baseline, we did not have the evolution of food preparation behaviors over the 5-year period, which could vary differently in subjects who became obese during the follow-up, compared with others. However, baseline food preparation behaviors of excluded obese individuals and participants who became obese were compared and no significant difference has been found (data not shown). This suggests that causal relationships between food preparation behaviors and risk to become obese is explained by differential exposure at baseline to specific dimensions of food preparation behaviors. Although the follow-up time was appropriate to perform our analyses, it did not necessarily guarantee this sufficient delay. In addition, this study was based on an observational cohort and thus residual confounding cannot be entirely ruled out even though a wide range of confounding factors were taken into account. Another limitation was that the data were self-reported, which may induce misreporting. Bias associated with social desirability is lower in studies using self-reported questionnaires, rather than face to face interviews, because it introduces distance between the investigator and the subject [81]. In addition, another study performed on a NutriNet-Santé cohort sample has demonstrated the validity of web-based self-reported anthropometric data by comparison with clinical data (*n* = 2513), and has shown that the reporting bias was reasonably small [82].

One strength of our study is the assessment of food preparation behaviors using an original questionnaire measuring various dimensions, while most previous studies have evaluated only one dimension. Food preparation behaviors are complex to define and measure [5, 15], therefore this questionnaire was carefully developed by experts, following the definition of one of the components of food literacy which fell into the “preparation” domain. This component includes being able to prepare commonly available foods, efficiently use common pieces of kitchen equipment and having a sufficient repertoire of skills to adapt recipes to experiment with food and ingredients [39]. An extensive literature review on different indicators such as frequency and time spent preparing food, self-estimated cooking skills and knowledge, enjoyment of cooking, preparation from scratch or complex food preparation techniques was performed to develop the questionnaire. However, the validity and reliability of the questions used to assess food preparation behaviors have not been explored. Although cooking skills and preparation from scratch showed adequate internal consistency, questions used here may not adequately capture these complex constructs, particularly cooking skills. Even though cooking skills regarding several common dishes, pastries and sweets, sauces and cooking techniques were assessed, they may be too specific to capture the full complexity of cooking skills. Also, people with different cooking skills may have responded similarly to the same questions because some people may prepare their meals from basic ingredients, while others may use ready to use foods. However, to avoid this bias, participants could report preparing the dishes entirely homemade or preparing the variant with ready for use ingredients. However, a comprehensive and validated measure of cooking skills is necessary for future studies. At last, external validity may be limited because this questionnaire was developed in a French cultural setting and cross-cultural adaptations may be required before submitting it to other cultures.

## Conclusions

In the unfavorable context from reduced time spent preparing meals over recent decades [1], our prospective study does not show effect of food preparation behaviors on relative weight change and obesity risk over 5 years, except for preparation from scratch. Our findings highlighted that preparation from scratch was associated with a decreased risk of obesity after 5 years of follow up in women and healthier dietary intake in women with greater preparation from scratch largely explained this relationship. Our study therefore provides useful information about the long term implications of food preparation behaviors on health and should be corroborated by future studies. In addition, as suggested by several reviews [16, 19, 40], there is a clear need for longitudinal studies to identify causal relationships, particularly to establish whether food preparation behaviors are associated with risk of chronic diseases such as incident diabetes, hypertension or cardiovascular diseases, compared with other determinants. Our findings emphasize the need to consider the use of fresh and minimally processed foods in the management and prevention of obesity. In addition to the health impact, the displacement of minimally processed foods and home prepared dishes and meals by ultra-processed products may be troublesome from social, cultural, economic, political and environmental points of view [49]. Further researches focusing on the impact of food preparation behaviors on these dimensions should be conducted.

## Additional file

Additional file 1: **Table S1** Repeatibility indicator of all items. **Table S2.** Computation of scores of food preparation behaviors. **Table S3.** Internal consistency of preparation from scratch and cooking skills. **Table S4.** Comparison of sociodemographic characteristics and dietary intake between the overall sample and the excluded subjects. (DOCX 28 kb)

## Abbreviation

BMI: Body Mass Index
