# Supplementary material for: Influence of food preparation behaviors on 5-year weight change and obesity risk in a French prospective cohort
Source: Int J Behav Nutr Phys Act. 2018 Nov 26;15:120. doi: 10.1186/s12966-018-0747-4 (PMC6258165; doi:10.1186/s12966-018-0747-4)
Supplement: Supplementary file 1 — Table S1 Repeatibility indicator of all items. Table S2. Computation of scores of food preparation behaviors. Table S3. Internal consistency of preparation from scratch and cooking skills. Table S4. Comparison of sociodemographic characteristics and dietary intake between the overall sample and the excluded subjects. (DOCX 28 kb) [file 12966_2018_747_MOESM1_ESM.docx]

Table S1. Repeatibility indicator of all items

| Dimension | Item | Prevalence- and bias-adjusted kappa coefficient |
| --- | --- | --- |
| Frequency and time for meal preparation | Frequency of preparation meals during a typical week | 0.43 |
|  | time spent preparing meals | 0.51 |
| Preparation from scratch | Grapefruit, orange, lemon, etc | 0.67 |
|  | Apple, pear, quince, etc | 0.62 |
|  | Apricot, peach, cherry, plum, etc | 0.64 |
|  | Grape, strawberry, raspberry, etc | 0.53 |
|  | Banana, pineapple, kiwi, mango, etc | 0.51 |
|  | Nuts | 0.44 |
|  | None fruit | 0.53 |
|  | Lettuce and other salad, endive, fresh spinach | 0.46 |
|  | Beets, carrots, celeriac, etc | 0.62 |
|  | Beans, peas, etc | 0.42 |
|  | Asparagus, celery, fennel, leeks, artichoke, etc | 0.44 |
|  | Tomato, pepper, eggplant | 0.62 |
|  | Cucumber, zucchini | 0.53 |
|  | Garlic, onions, shallots | 0.55 |
|  | Broccoli, cauliflower, cabbage, Brussels sprouts, etc | 0.56 |
|  | Mushrooms | 0.38 |
|  | Potatoes | 0.57 |
|  | None vegetable | 0.51 |
|  | Whole fish not cleaned out | 0.38 |
|  | Whole fish cleaned out | 0.45 |
|  | Fish fillets, sliced, pavers or steaks | 0.59 |
|  | Breaded fish | 0.53 |
|  | None form of fish | 0.59 |
|  | chunky uncut pieces | 0.37 |
|  | whole poultry not cleaned out | 0.35 |
|  | whole poultry cleaned out | 0.48 |
|  | cut poultry or meat | 0.43 |
|  | ready to cook poultry or meat | 0.46 |
|  | None form of poultry or meat | 0.47 |
| Cooking skills | Make bread | 0.56 |
|  | Make mashed potatoes | 0.60 |
|  | Make savory pie or pizza | 0.57 |
|  | Make a vegetable gratin | 0.48 |
|  | Make a dish with stewed meat or fish | 0.50 |
|  | Make ice cream or sorbet | 0.40 |
|  | Make yogurt | 0.32 |
|  | Make pancakes or waffles | 0.46 |
|  | Make cakes or pastries | 0.44 |
|  | Make floating islands | 0.51 |
|  | Make chocolate mousse | 0.47 |
|  | Make macaroons | 0.43 |
|  | Make pie | 0.44 |
|  | Make salad dressing | 0.55 |
|  | Make mayonnaise | 0.46 |
|  | Make garlic butter | 0.40 |
|  | Make bechamel | 0.42 |
|  | Make tomato sauce | 0.60 |
|  | Make hollandaise sauce | 0.43 |
|  | Make sauce by reduction | 0.40 |
|  | None sauce | 0.59 |
|  | Scale and clear out a whole fish | 0.45 |
|  | Fillet a whole fish | 0.51 |
|  | Stuff meat or poultry | 0.55 |
|  | Tie up a roast | 0.40 |
| Kitchen equipment | pressure cooker | 0.51 |
|  | zester | 0.44 |
|  | baking pan | 0.49 |
|  | measuring cup | 0.41 |
|  | food processor | 0.64 |
|  | gas oven or electric oven | 0.61 |
| Enjoyment | Enjoyment for food preparation | 0.55 |
|  | Willingness to cook better | 0.44 |
|  | Willingness to cook more frequently | 0.48 |

Table S2. Computation of scores of food preparation behaviors

| **Use of foods with no or minimal processing (from 0 to 12 points)** | Points |
| --- | --- |
| *Unpeeled, uncut, unprocessed vegetables* |  |
| Lettuce and other salad, endive, fresh spinach | 1 |
| Beets, carrots, celeriac, etc | 1 |
| Beans, peas, etc | 1 |
| Asparagus, celery, fennel, leeks, artichoke, etc | 1 |
| Broccoli, cauliflower, cabbage, Brussels sprouts, etc | 1 |
| Mushrooms | 1 |
| *Forms of fish used* |  |
| Use of whole fish not cleaned out (even if participant also used whole fish cleaned out or fish fillets, sliced, pavers or steaks) | 2 |
| Use of whole fish cleaned out (even if participant also used fish fillets, sliced, pavers or steaks) | 2 |
| Breaded fish (even if participant also used whole fish not cleaned out, whole fish cleaned out or fish fillets, sliced, pavers or steaks) | 1 |
| Breaded fish only or none | 0 |
| *Forms of meat used* |  |
| Chunky uncut pieces, whole poultry not cleaned out, whole poultry cleaned out, cut poultry or meat (even if participant also used ready to cook poultry or meat) | 1 |
| Ready to cook poultry or meat only (ultra-processed met and nuggets) or none | 0 |
| **Cooking skills (from 0 to 41 points)** |  |
| *Dishes* |  |
| Make bread | 2 |
| Make bread with a bread maker | 1 |
| Make mashed potatoes (with unprocessed potatoes) | 2 |
| Make instant mashed potatoes | 1 |
| Make savory pie or pizza with homemade pastry shell | 2 |
| Make savory pie or pizza with ready for use pastry shell | 1 |
| Make a vegetable gratin | 1 |
| Make a dish with stewed meat or fish | 1 |
| *Desserts and pastries* |  |
| Make ice cream or sorbet with or without ice cream maker | 1 |
| Make yogurt with or without yogurt-maker | 1 |
| Make pancakes or waffles with homemade pancake batter | 2 |
| Make pancakes or waffles with ready for use pancake batter | 1 |
| Make cakes or pastries with homemade pastry shell | 2 |
| Make cakes or pastries with ready for use pastry shell | 1 |
| Make floating islands with homemade custard | 2 |
| Make floating islands with ready for use custard | 1  1 |
| Make chocolate mousse | 1 |
| Make macaroons | 1 |
| Make pie with homemade pastry shell | 2 |
| Make pie with ready for use pastry shell | 1  1 |
| *Sauces* |  |
| Make hollandaise sauce or sauce by reduction (even if participant can make or not salad dressing) | 4 |
| Make 3 or 4 simple sauces* (even if participant can make or not salad dressing) | 3 |
| Make 2 simple sauces (even if participant can make or not salad dressing) | 2 |
| Make only 1 simple sauce (even if participant can make or not salad dressing) | 1 |
| Make salad dressing or none | 0 |
| *Cooking techniques* |  |
| Scale and clear out a whole fish | 1 |
| Fillet a whole fish | 1 |
| Stuff meat or poultry | 1 |
| Tie up a roast | 1 |
| **Kitchen equipment (from 0 to 11 points)** |  |
| Have pressure cooker | 2 |
| Have zester | 2 |
| Have baking pan | 2 |
| Have measuring cup | 2 |
| Have food processor | 2 |
| Have gas oven or electric furnace | 1 |

*Simple sauces: mayonnaise, garlic butter, bechamel, tomato sauce

Table S3. Internal consistency of preparation from scratch and cooking skills

| Dimension | Item | Item-factor r^1^ | Ordinal Cronbach's  Alpha |
| --- | --- | --- | --- |
| Preparation from scratch | Unpeeled, uncut, unprocessed vegetables | 0.77 | 0.72 |
|  | Forms of fish used | 0.60 |  |
|  | Forms of meat used | 0.47 |  |
| Cooking skills | Make bread | 0.52 | 0.84 |
|  | Make mashed potatoes | 0.67 |  |
|  | Make savory pie or pizza | 0.72 |  |
|  | Make a vegetable gratin | 0.42 |  |
|  | Make a dish with stewed meat or fish | 0.45 |  |
|  | Make ice cream or sorbet | 0.68 |  |
|  | Make pancakes or waffles | 0.70 |  |
|  | Make cakes or pastries | 0.74 |  |
|  | Make floating islands | 0.65 |  |
|  | Make chocolate mousse | 0.52 |  |
|  | Make macaroons | 0.43 |  |
|  | Make pie | 0.69 |  |
|  | Make sauces | 0.71 |  |
|  | Scale and clear out a whole fish | 0.45 |  |
|  | Fillet a whole fish | 0.51 |  |
|  | Stuff meat or poultry | 0.55 |  |
|  | Tie up a roast | 0.41 |  |

1 Polychoric correlations between each studied item and its assigned dimension corrected for overlap (i.e. the dimension is modiﬁed by excluding the studied item)

Table S4. Comparison of sociodemographic characteristics and dietary intake between the overall sample and the excluded subjects

|  | **Overall sample (n=79,960)**  **% or mean (SD)** | **Excluded subjects**  **(n=67,109)**  **% or mean (SD)** | **P-value^1^** |
| --- | --- | --- | --- |
|  |  |  |  |
| **Sex** |  |  |  |
| Men | 22.4 | 16.6 | 0.0001 |
| Women | 77.6 | 83.4 |  |
|  |  |  |  |
| **Age** |  |  |  |
| 18-24 years | 5.2 | 8.3 | 0.002 |
| 25-34 years | 19.7 | 28.6 |  |
| 35-54 years | 39.3 | 40.9 |  |
| > 55 years | 35.8 | 22.2 |  |
|  |  |  |  |
| **Education** |  |  |  |
| Primary | 3.1 | 4.4 | 0.08 |
| Secondary | 33.3 | 32.3 |  |
| Under-graduate | 30.1 | 31.3 |  |
| Post-graduate | 33.5 | 32.0 |  |
|  |  |  |  |
| **Occupation** |  |  |  |
| Never employed | 4.4 | 6.7 | 0.05 |
| Self-employed | 3.2 | 3.1 |  |
| Manual worker, office worker | 30.3 | 38.2 |  |
| Intermediate profession | 27.6 | 27.3 |  |
| Managerial staff | 34.5 | 24.7 |  |
|  |  |  |  |
| **Monthly household income per household unit** |  |  |  |
| Unwilling to answer | 10.8 | 14.4 | 0.003 |
| < 1200 euros | 15.3 | 19.3 |  |
| 1200-1800 euros | 24.7 | 11.4 |  |
| 1801-2700 euros | 24.3 | 37.9 |  |
| > 2700 euros | 24.9 | 17.0 |  |
|  |  |  |  |
| **Household composition** |  |  |  |
| Single | 17.1 | 18.6 | 0.11 |
| Couple without child | 41.2 | 35.7 |  |
| Couple with ≥one child | 31.5 | 35.0 |  |
| Household without child and with ≥3 adults | 10.2 | 10.7 |  |
|  |  |  |  |
| **Physical activity level** |  |  |  |
| Low | 34.2 | 29.8 | 0.005 |
| Moderate | 48.2 | 54.5 |  |
| High | 17.6 | 15.7 |  |
|  |  |  |  |
| **Smoking status** |  |  |  |
| Never-smoker | 50.2 | 53.0 | 0.05 |
| Former smoker | 35.2 | 29.0 |  |
| Current smoker | 14.6 | 18.0 |  |
|  |  |  |  |
| **Dieting to lose weight** |  |  |  |
| Never dieter | 21.6 | 18.2 | 0.03 |
| Former dieter | 63.2 | 60.4 |  |
| Current dieter | 15.2 | 21.4 |  |
|  |  |  |  |
| **Energy intake** | 1936.7 (515.9) | 1805.3 (555.7) | 0.03 |
| **Fruit and vegetable intake** | 444.8 (285.8) | 402.8 (159.1) | 0.05 |
| **Red and processed meat intake** | 61.6 (64.8) | 68.3 (60.1) | 0.11 |
| **Alcoholic beverages intake** | 104.2 (155.0) | 132.3 (148.6) | 0.01 |
|  |  |  |  |
|  |  |  |  |

1 P-value represented the overall significance of each variable
